# A psychobiological resilience factor for mental health across the lifespan

**DOI:** 10.21203/rs.3.rs-7861905/v1

**Published:** 2025-10-29

**Authors:** Tianye Jia, Zhengyu Yang, Shitong Xiang, Rongquan Zhai, Chen Zheng, Yechen Hu, Tobias Banaschewski, Arun Bokde, Sylvane Desrivières, Herta Flor, Hugh Garavan, Penny Gowland, Antoine Grigis, Andreas Heinz, Jean-Luc Martinot, Marie-Laure Martinot, Eric Artiges, Frauke Nees, Dimitri Papadopoulos Orfanos, Luise Poustka, Michael Smolka, Sarah Hohmann, Nathalie Holz, Nilakshi Vaidya, Henrik Walter, Robert Whelan, Gunter Schumann, Jianfeng Feng, Barbara Sahakian, Chao Xie

**Affiliations:** Fudan University; Institute of Science and Technology for Brain-inspired Intelligence, Fudan University; Institute of Science and Technology for Brain-Inspired Intelligence, Fudan University; Fudan University; Fudan University; Institute of Science and Technology for Brain-Inspired Intelligence, Fudan University; Central Institute of Mental Health, Mannheim; Discipline of Psychiatry, School of Medicine and Trinity College Institute of Neuroscience, Trinity College Dublin, Dublin, Ireland; Institute of Psychiatry, Psychology & Neuroscience, King’s College London, United Kingdom; Central Institute of Mental Health Medical Faculty Mannheim Heidelberg University; Departments of Psychiatry and Psychology, University of Vermont, 05405 Burlington, Vermont, USA; University of Nottingham; Universität Berlin; Institut National de la Santé et de la Recherche Médicale; Institut National de la Santé et de la Recherche Médicale, INSERM U 1299; INSERM/Université Paris-Saclay; Central Institute of Mental Health; Department of Child and Adolescent Psychiatry and Psychotherapy, University Medical Centre Gottingen; Technische Universität Dresden; Technische Universität Dresden; Heidelberg University; Charité - Universitätsmedizin Berlin; University Medical Centre Charité; Trinity College; Charite Universitaetsmedizin Berlin; Institute of Science and Technology for Brain Inspired Intelligence, Fudan University, Shanghai, PR China; University of Cambridge; Institute of Science and Technology for Brain-Inspired Intelligence, Fudan University

## Abstract

Psychological resilience plays a crucial role in maintaining individual mental health in the face of stressful events, adversity, and even trauma. However, resilience, may be a dynamic and evolving process, which presents challenges for precise definition and measurement, particularly during the rapid developmental period of adolescence. In this study, we demonstrated that the self-reported ability to bounce back from adversity of middle-aged to older adults (UKB) can reflect their mental fortitude over a 16-year period. We then constructed a psychosocial model based on UKB to predict resilience from multi-level factors, such as personality traits, family-support, and economics. The model revealed that personality traits and life satisfaction accounted for the largest proportion of resilience variance. When applied prospectively to a longitudinal adolescent cohort (IMAGEN; n=692), this model accurately identified trauma-exposed adolescents who maintained mental health outcomes comparable to non-traumatised peers up to age 23. These findings demonstrate that psychosocial mechanisms stabilised in adulthood can predict adaptive functioning after early adversity, providing a lifespan framework for the early identification and prevention of mental health vulnerability.

## Introduction

Exposure to life adversity imposes immense stress on individuals, substantially increasing the risk of disruptions to both psychological and physiological functions^1^. However, individuals exhibit substantial variation in their responses to adversity, with many maintaining psychological well-being despite severe life events, a phenomenon known as resilience^2–5^. These empirical findings contribute to understanding the psychosocial mechanisms by which people overcome adversity and inform clinical strategies for the early intervention and treatment of stress-related disorders, such as post-traumatic stress disorder, depression, and anxiety^6,7^.

Individuals who remain unaffected or recover quickly from psychological disorders after adversity are considered resilient, which is referred to as an outcome-based approach^8^. However, a recent study using this empirical approach found that the protective effect of resilience might diminish from adolescence to adulthood. Specifically, individuals who experienced childhood trauma but remained free of psychiatric diagnoses by age 16 were still at a higher risk for delayed, poorer outcomes emerging in early adulthood^9^. This finding raised concerns about the construct validity of outcome-based identification of resilience, particularly during adolescence^9–12^, which is characterised by rapid neurocognitive development and unstable mental states. Nevertheless, adolescence is also a crucial period for mental health development, as it marks the peak onset of many psychiatric disorders^13^. In this context, early intervention for individuals at potential risk due to adversity is critical to prevent the emergence of psychological dysfunction during adolescence^14^. Therefore, improving the precise identification of resilience could offer a deeper understanding of the long-term trajectories of individuals exposed to adversity, and enable timely and targeted prevention interventions, thereby improving public health.

**Figure 1:**
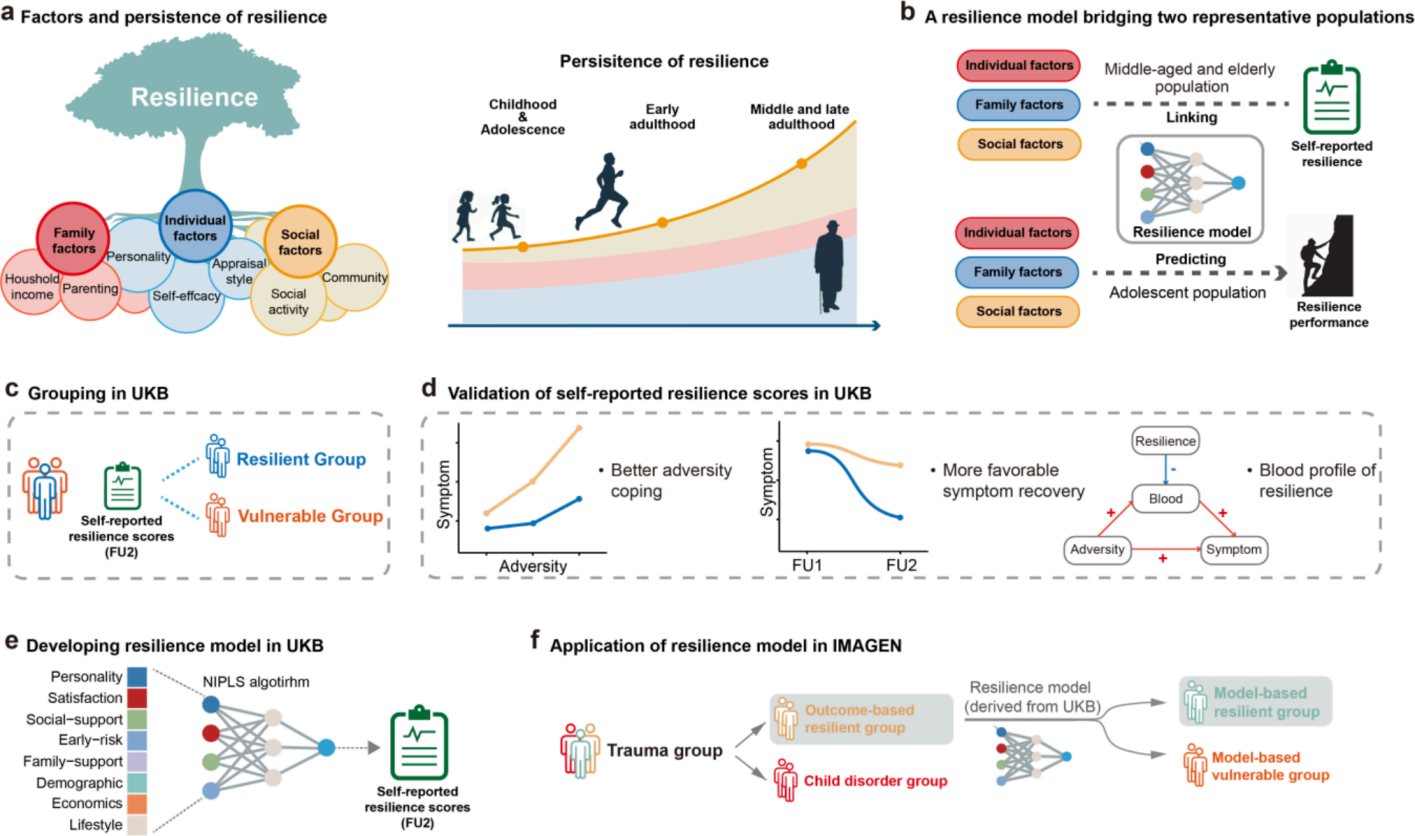
Overview of research farmwork. (a) Illustration of the link between resilience factors and resilience outcomes, with individual persistence of resilience increasing with age as resilience factors stabilize, as shown in the trend. (b) Illustration of a resilience model constructed from psychosocial factors in middle-to-late adults (UK Biobank), leveraging their higher resilience persistence and stable mechanisms, and its application to predict resilience outcomes in adolescents (IMAGEN). (c) Resilience scores from the Brief Resilience Scale (BRS) in the UK Biobank dataset were obtained from self-reports at the final time point. Individuals were categorized into resilient (top 33%) and vulnerable (bottom 33%) groups based on these scores. (d) Validation of the BRS in the UK Biobank dataset through retrospective analysis, examining performance under adversity, recovery from psychological symptoms, and stress-related physiological responses in relation to BRS scores. (e) We developed a psychosocial model to link multi-level factors (including individual, family, and community) with individual BRS scores, aiming to explore the psychosocial mechanisms of resilience and predict individual resilience outcomes. (f) We applied the resilience model developed from the UKB to an adolescent cohort, proposing a model-based method for defining resilience with the aim of more accurately identifying resilient individuals.

Resilience is increasingly viewed as a constructive process, rather than a static outcome, shaped by multi-level factors including individual characteristics, family, and community^8,12,15^. This process may evolve over time following adversity and through ongoing interactions with the external environment, and becomes stabilised from a certain age^16^. However, the majority of research in this field has primarily used single-variable approaches to study resilience-related factors, which may overlook their complex interactions^17^. Meanwhile, it should also be noted that the dynamic nature of resilience complicates accurate identification of factors underlying it, especially during the rapid developmental periods of childhood and adolescence. For instance, while the consistent associations between personality traits and resilience have been reported in adult cohorts, studies in adolescents have revealed inconsistent associations^18,19^. These observations suggest that adulthood, with more stable psychological and physiological functions, established socioeconomic status, and accumulated life experiences, may provide a more suitable context for investigating the psychosocial factors underlying resilience (Fig. 1a).

Therefore, we proposed a lifespan framework of modelling resilience by investigating psychosocial factors in middle-to-late adults (UK Biobank, UKB) and applying this model to accurately identify individuals who can maintain mental health well-being following trauma events in adolescence (IMAGEN) (Fig. 1b). In this study, we first examined whether the resilience of middle-to-late adults in the UKB cohort can be effectively measured using a self-report scale (Brief Resilience Scale, BRS), e.g., retrospectively predictive for better mental health well-being over the past decades (Fig. 1c, d). Then, we used a nonlinear iterative partial least squares (NIPLS) algorithm to model individuals’ self-reported resilience scores with multi-level factors (individual, family, and community levels) and evaluate each factor’s contribution (Fig. 1e). Finally, in a large adolescent cohort, where an outcome-based identification of resilience is likely to fail^9^, we aimed to accurately identify individuals who will show long-term resilient performance by integrating the established computational psychological model and the outcome-based model of resilience (Fig. 1f). In summary, this lifespan approach integrates outcome-based and process-based perspectives to enhance our understanding of the psychosocial mechanisms of resilience and advance the development of preventive and interventional strategies for adversity-related disorders.

## Results

### Relationship between resilience, trauma exposure, and mental health

**Figure 2:**
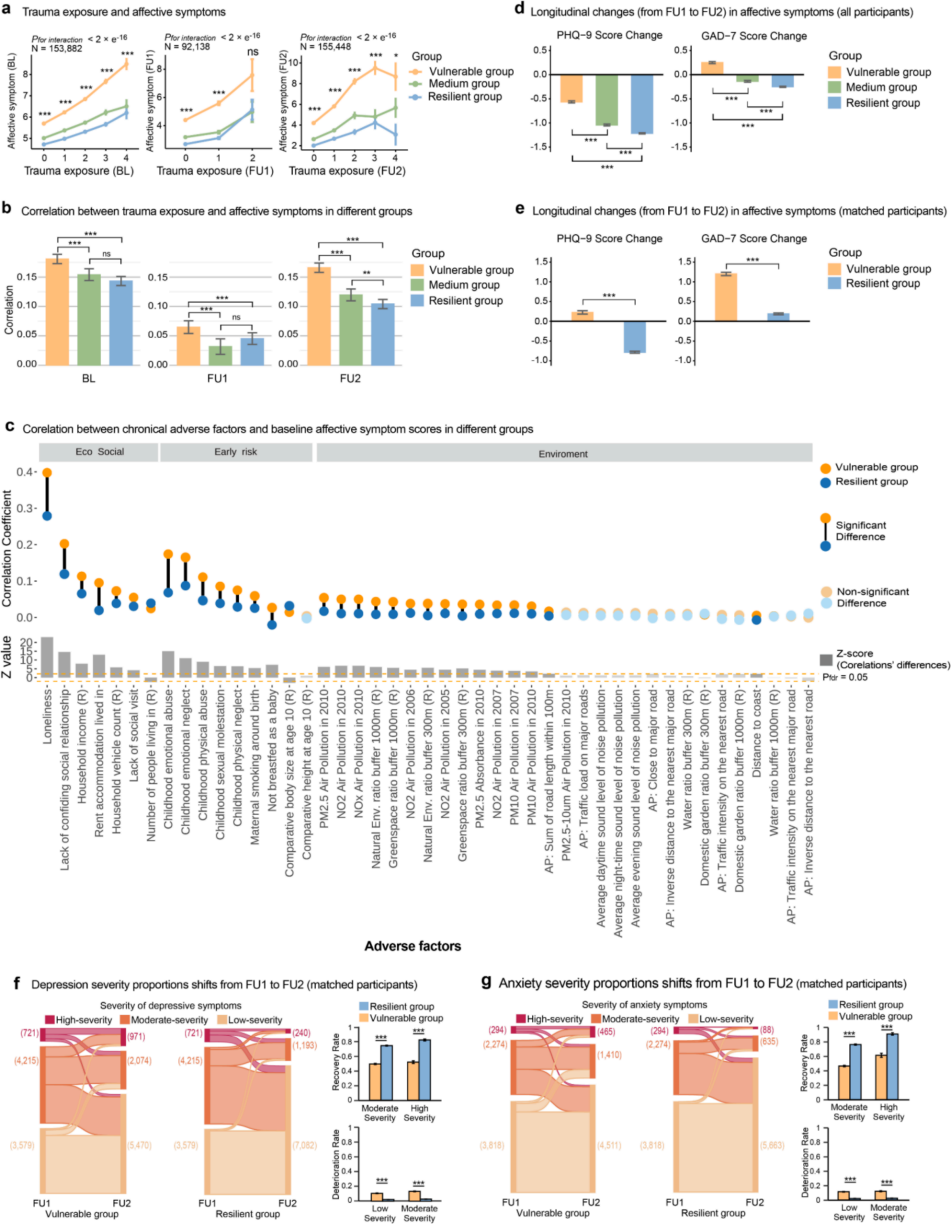
Impact of trauma exposure on affective symptoms across resilience groups and longitudinal changes in symptom severity. (a) Line plot of affective symptom scores (BL: PHQ-4; FU1, FU2: PHQ-9 + GAD-7) by trauma exposure levels, with individuals grouped by resilience scores; resilient group showed the smallest symptom increase. (b) Bar plot of correlation coefficients (error bars: 95% CI) between trauma exposure and affective symptoms (BL, FU1, FU2) across resilience groups; resilient group had weaker associations than vulnerable group. (c) Dumbbell plot of correlations between general adverse factors and affective symptoms at BL in resilient and vulnerable groups; significant differences (*p*_*fdr*_<0.05) are marked. (d) Line plot of affective symptom scores (PHQ-9 + GAD-7) at FU1 and FU2; resilient group showed greater symptom reduction. (e) Line plot of affective symptom scores at FU1 and FU2 in resilient and vulnerable groups, matched for covariates (age, sex, ethnicity, education, BMI, FU1 PHQ-9, GAD-7) to ensure identical profiles. (f) Left: Sankey diagram of depression severity (PHQ-9) changes from FU1 to FU2 in resilient and vulnerable groups, matched for covariates and FU1 scores. Right: Bar plot of recovery and worsening proportions by FU1 depression severity. (g) Left: Sankey diagram of anxiety severity (GAD-7) changes from FU1 to FU2 in resilient and vulnerable groups, matched for covariates and FU1 scores. Right: Bar plot of recovery and worsening proportions by FU1 anxiety severity. Error bars represent mean ± SE, and significant differences are marked as * *p*<0.05, ** *p*<0.01, *** *p*<0.001.

In the UK Biobank (UKB), 164,929 participants (mean age 55.41 years at baseline [SD 7.51, range 39–72]; 95,195 [57.7%] female; 160,347 [97.2%] White) with complete baseline covariates completed the Brief Resilience Scale (BRS) at follow-up 2 (FU2). Given that resilience was characterized as maintaining mental health after adversities, we conducted retrospective analyses of the psychological states of the elderly population in the UKB when they were actually confronted with adversities, to validate the validity and longitudinal reliability of the self-reported BRS scores in measuring resilience. We observed trauma exposure was significantly associated with higher psychiatric symptoms across multiple time points (BL: *r* = 0.16, *P*_*two-tailed*_ < 1.0 × 10^−323^, N = 92,510; FU1: *r* = 0.05, *P*_*two-tailed*_ = 7.80 × 10^−58^, N = 92,510; FU2: *r* = 0.15, *P*_*two-tailed*_ < 1.0 × 10^−323^, N = 92,510). We classified participants into three groups based on their resilience scores: the resilient group (≥ 66th percentile, Brief Resilience Scale score ≥ 4, N = 63,741), the moderate group (33rd–66th percentile, 3.33 < score < 4, N = 38,395), and the vulnerable group (≤ 33rd percentile, score ≤ 3.33, N = 62,793), to examine whether the resilient group maintains better psychological well-being when facing trauma (group characteristics are detailed in **Supplementary Table 8**). Indeed, we found that the resilient group experienced the least negative impact when facing trauma, as demonstrated by the lowest positive association between trauma exposure and affective symptoms compared to other groups across multiple time points (Fig. 2a, 2b, resilient group compared to the vulnerable group: BL: *Z* = −4.00, *P*_*two-tailed*_ = 6.24 × 10^−5^; FU1: *Z* = −2.03, *P*_*two-tailed*_ = 4.19 × 10^−2^; FU2: *Z* = −8.29, *P*_*two-tailed*_ < 1.0 × 10^−323^). We also conducted subgroup analyses stratified by sex and in sensitivity analyses using continuous resilience scores, yielding similar results (**Supplementary Table 9–12**). These findings suggest that individuals with higher self-reported BRS scores have indeed demonstrated a sustained ability to cope with trauma over the past decade, experiencing less adverse impact on their psychological well-being.

As traumatic events were typically regarded as acute stressors, we further investigated the capacity of individuals classified within the resilient group to effectively manage a wider range of enduring adverse circumstances. We collected a wide range of long-term adverse factors across three domains (45 phenotypes, 7 in socioeconomic environment, 9 in early-life risks, and 30 in natural environment). At baseline, we found that most long-term adverse factors were correlated with poorer mental health (37/45 adverse factors, *P*_*FDR*_ < 0.05, **Supplementary Table 13**). Furthermore, individuals in the resilient group experienced less detrimental effects on their mental health in response to adverse factors, indicated by significantly weaker associations between most of these adverse factors and affective symptoms compared to those in the vulnerable group (27/37, *P*_*FDR*_ < 0.05, Fig. 2c). This protective effect was persistent across FU1 and FU2 (FU1: where 37/45 adverse factors affecting mental health negatively, the resilient group was less affected by 19 factors (51.4%), **Supplementary Table 14, Supplementary Fig. 1a**; FU2: where 37/45 adverse factors affecting mental health negatively, the resilient group showed lesser negative effects in 21 factors (56.8%), **Supplementary Table 15, Supplementary Fig. 1b**). These findings further indicated that individuals with higher BRS scores possess a greater capacity to cope with various adverse factors in their lives, reinforcing the validity of the BRS as a measure of resilience among middle-to-older adults. Since the BRS focuses on an individual’s self-perceived ability to bounce back from stress, we proposed that individuals in this age range, drawing from their extensive life experiences, may be better equipped to accurately assess their own resilience.

### Resilience and the longitudinal changes in mental health

From a longitudinal perspective, we checked the trajectory across FU1 and FU2 which used the same questionnaire (N = 103,799). We found that the resilient group showed greater reductions of affective symptom scores from FU1 to FU2 (reduction difference in PHQ scores: *β* = −0.66, 95% CI = −0.71 to −0.62, *T* = −29.41, *Cohen’ d* = −0.20, *P*_*two-tailed*_ = 2.15 × 10^−189^, **Supplementary Table 16**; reduction difference in GAD scores: *β* = −0.52, 95% CI = −0.57 to −0.48, *T* = −23.78, *Cohen’ d* = −0.16, *P*_*two-tailed*_ < 1.25 × 10^−124^, **Supplementary Table 17**; Fig 2d,). This effect was even stronger after we regressed the individuals’ affective symptoms scores at FU1 (reduction difference in PHQ scores: *β* = −1.66, 95% CI = −1.70 to −1.62, *T* = 79.80, *Cohen’ d* = −0.49, *P*_*two-tailed*_ < 1.0 × 10^−323^, **Supplementary Table 16**; reduction difference in GAD scores: *β* = −1.65, 95% CI = −1.68 to −1.61, *T* = −84.37, *Cohen’ d* = −0.52, *P*_*two-tailed*_ < 1.0 × 10^−323^, **Supplementary Table 17**). In addition, we employed a sample-matching approach, propensity-score matching (PSM) to balance psychiatric symptom scores at FU1, as well as covariates such as age, gender, BMI, ethnicity, and education, between the resilient and vulnerable groups (N = 7,724, for balanced characteristics of the resilient and vulnerable groups at FU1, see **Supplementary Table 18**). We also found the individuals in the resilient group exhibited greater decreases in affective symptom scores compared to those in the vulnerable group (reduction difference in PHQ scores: *β* = −1.23, 95% CI = −1.31 to −1.15, *T* = −20.06, *Cohen’s d* = −0.46, *P*_*two-tailed*_ = 2.63 × 10^−193^; reduction difference in GAD scores: *β* = −1.34, 95% CI = −1.40 to −1.29, *T* = −20.80, *Cohen’s d* = −0.47, *P*_*two-tailed*_ < 1.0×10^−323^, Fig 2e, **Supplementary Table 19–20**, Fig. 2e). These findings indicated that individuals characterized by high resilience scores displayed a more favourable mental health outcome over time, irrespective of their mental health condition at the FU1.

We further classified individuals into three levels of symptom severity for depression (PHQ-9: high >14, moderate 10–14, low <10) and anxiety (GAD-7: high >14, moderate 10–14, low <10) to track changes in clinical symptoms across the two visits^20,21^. We matched the resilient and vulnerable groups within each symptom severity level by PSM to balance the number of participants, PHQ/GAD scores, and covariates across the two groups (**Supplementary Table 21–22**). We found that the resilient group, compared to the vulnerable group, showed a higher recovery rate from moderate- and high-severity symptom states for both depression and anxiety (83.9% vs. 53.0% recovery from high-severity depression, OR = 4.61, 95% CI = 3.61 to 5.93, *P*_*two-tailed*_ = 1.33 × 10^−36^; 75.9% vs. 50.4% recovery from moderate-severity depression, OR = 3.10, 95% CI = 2.82 to 3.40, *P*_*two-tailed*_ = 2.43 ×10^−130^, Fig 2f; 91.2% vs. 61.6% recovery from high-severity anxiety, OR = 6.38, 95% CI = 4.06 to 10.38, *P*_*two-tailed*_ = 3.05 ×10^−17^; 76.4% vs. 46.6% recovery from moderate-severity anxiety, OR = 3.71, 95% CI = 3.27 to 4.21, *P*_*two-tailed*_ = 7.43×10^−95^, Fig 2g). This suggests that the resilience measured by the BRS not only aids in coping with adversity but also supports broader psychological well-being. We proposed that this may stem from individuals with higher BRS scores being better equipped to manage everyday hassles.

### Protective role of resilience on biological processes in trauma exposure

Traumatic events, as acute stressors, are known to elicit a cascade of physiological changes implicated in affective disorders^22–25^. Using routine blood test results (blood count and blood biochemistry, N = 12,394–148,537 per specific blood parameters) from the UKB dataset, we identified 32 out of 61 blood parameters significantly associated with both trauma exposure and affective disorder at BL (*P*_*FDR*_ < 0.05, **Supplementary Table 23**). Of these, 25 exhibited significant mediation effects on the relationship between trauma exposure and affective disorders (*P* < 0.001, **Supplementary Table 24**), suggesting that trauma-related physiological alterations may contribute to affective symptoms via a psychophysiological pathway.

**Figure 3:**
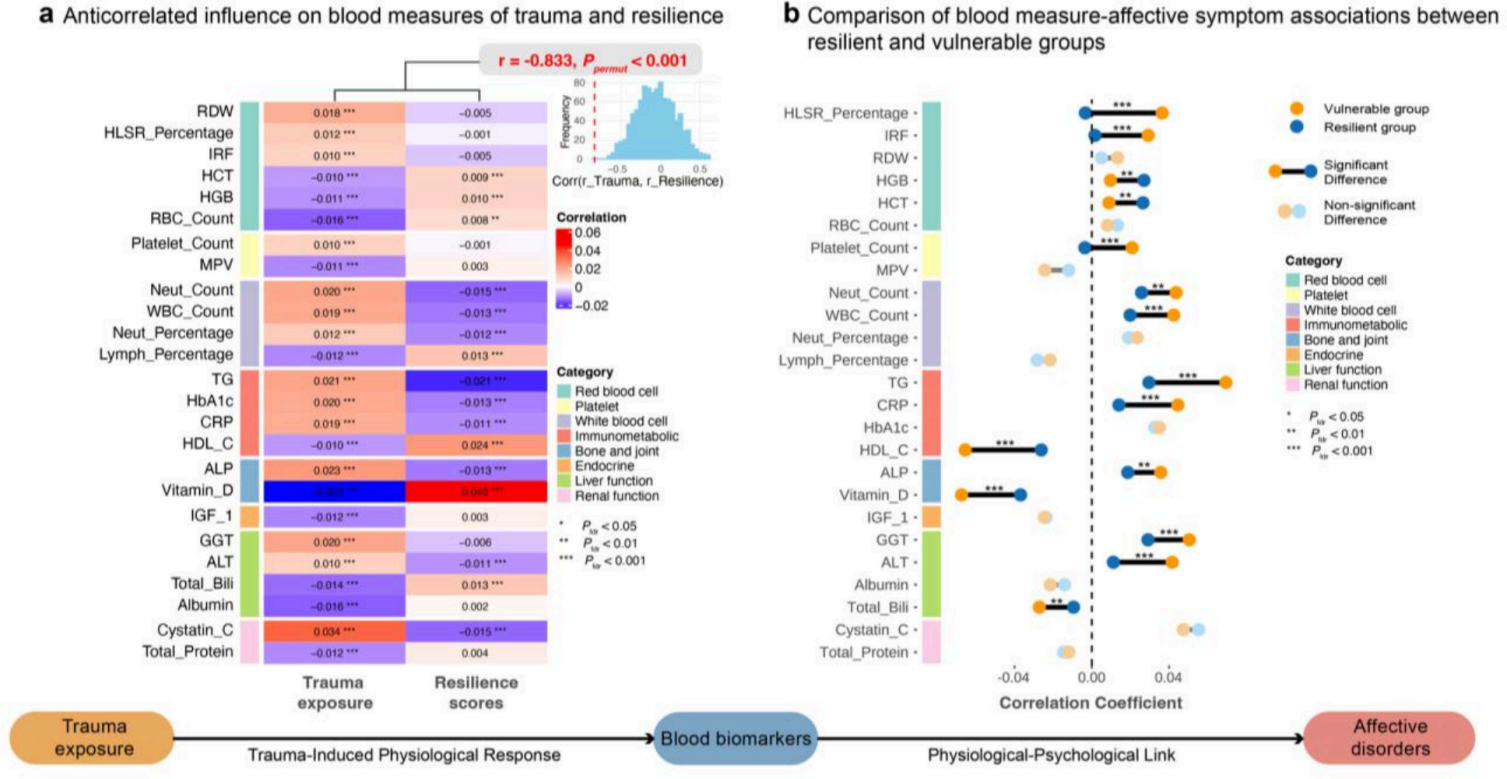
The protective role of resilience on immune-related blood biomarkers admits trauma exposures. (a) Heatmap displaying the partial correlations of 25 blood biomarkers, significantly mediating the relationship between trauma exposure and affective disorders (*p*<0.001), with trauma exposure and resilience scores. Covariates (age, sex, site, education years, and BMI) were controlled. Of these, 15 biomarkers overlapped, showing opposite correlation directions between trauma exposure and resilience. The correlation pattern between the 25 biomarkers’ associations with trauma and resilience was inverse, with a correlation coefficient of r=−0.833 and *p*<0.001, determined by a permutation test with 1000 iterations. (b) Bar plot comparing the correlations between the same 25 mediating blood biomarkers and affective symptoms (PHQ scores) in resilient and vulnerable groups, illustrating resilience’s moderating effect. Among these, 13 biomarkers showed significantly weaker correlations with affective symptoms in the resilient group compared to the vulnerable group (*p*_*fdr*_<0.05).

Among these 25 mediating blood parameters, we observed substantial overlap with biomarkers associated with resilience scores assessed at follow-up 2 (FU2). Specifically, 15 were significantly correlated with resilience (*P*_*FDR*_ < 0.05), including 8 immune-related markers, 3 red blood cell-related indicators, 2 bone and joint-related markers, 2 liver function-related parameters, and 1 renal function-related marker, with correlation directions consistently opposite to those with trauma exposure (Fig 3a). A permutation test (1,000 iterations) on these 25 mediators confirmed this inverse pattern, showing a strong negative correlation (*r* = −0.82, *P*_*permut*_ < 0.001) between their associations with trauma and resilience, suggesting a distinct physiological profile—particularly influenced by immune-related factors—underlying resilience compared to trauma-related vulnerability.

Furthermore, within the trauma-to-affective disorder pathway, resilience moderates the relationship between these mediating blood parameters and affective symptoms. Regression analyses revealed that for 22 of the 25 mediators, higher resilience attenuated their association with affective outcomes, as evidenced by significant interaction terms (beta coefficients opposite to main effects, *P*_*FDR*_ < 0.05, **Supplementary Table 25**). This moderation was supported by comparing correlations with affective symptoms across resilient and vulnerable groups, with 13 mediators showing significantly weaker associations in the resilient group (*P*_*FDR*_ < 0.05, Fig 3b, **Supplementary Table 26**). These findings may represent a mechanism by which psychological fortitude mitigates the adverse effects of physical dysregulation on emotional well-being.

Similarly, using UK Biobank plasma proteomics data (2,920 proteins, N = 11,507–15,833 per specific proteins), we identified 72 proteins mediating the trauma-to-affective disorder pathway (**Supplementary Table 27**, *P* < 0.001). The Gene Ontology (GO) Biological Process (BP) enrichment and Reactome pathway enrichment analyses of these proteins predominantly revealed immune-related processes (**Supplementary Table 29–31**, **Supplementary Fig 2b, c**). Of these, 10 overlapped with resilience-associated proteins (*P*_*FDR*_ < 0.05), displaying an opposite correlation pattern between trauma and resilience associations (*r* = −0.617, *P*_*permut*_ < 0.001, **Supplementary Fig 2a**), consistent with previous findings. Resilience also moderated the relationship between 52 of these 72 mediators and affective disorders (*P*_*FDR*_ < 0.05, **Supplementary Table 28**), reinforcing its protective role across physiological measures.

### Resilience model in aged cohort

**Figure 4:**
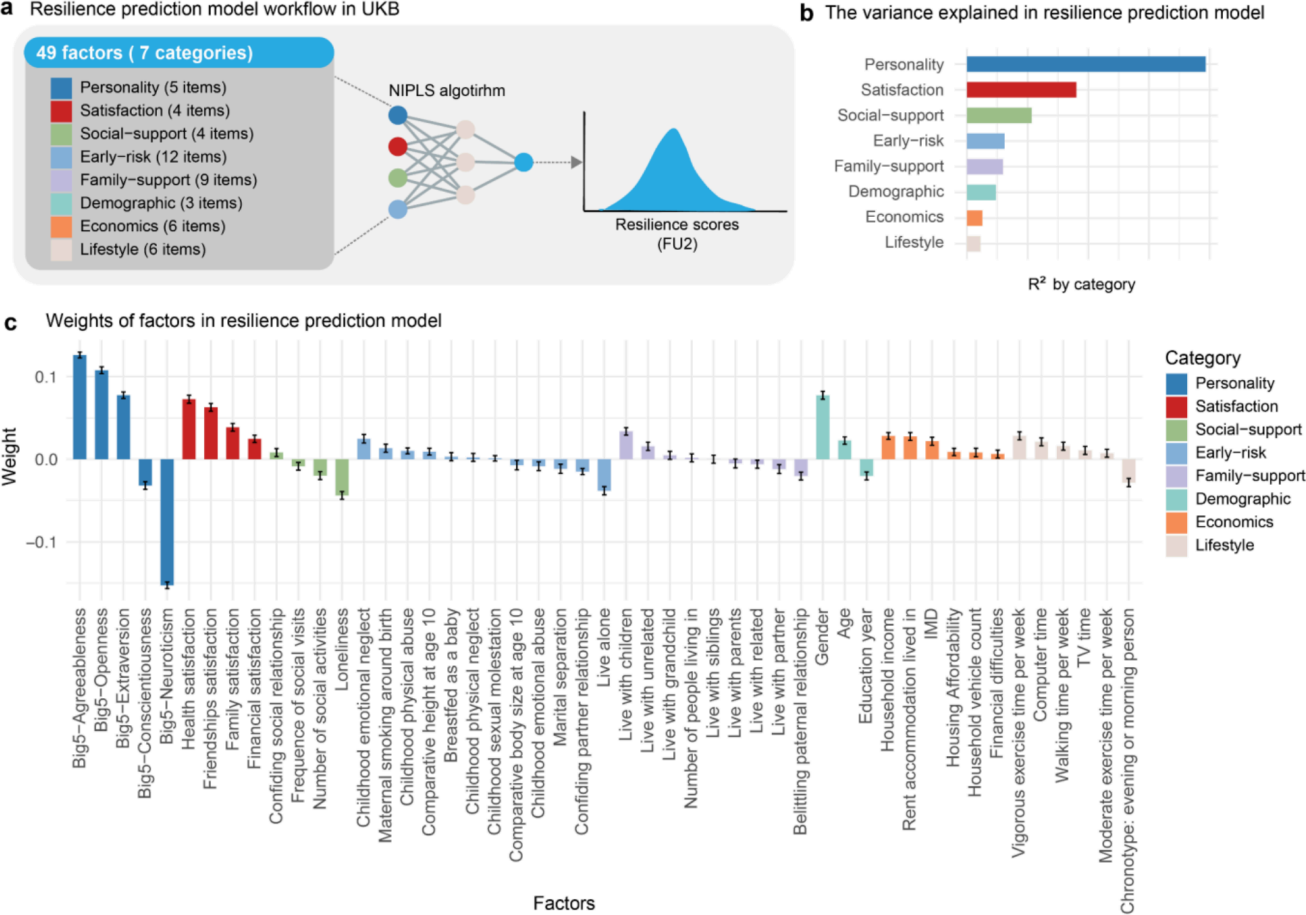
A behaviour model based on the UKB dataset. (a) Schematic showing the predictive model workflow. 49 factors grouped into 7 categories were used to predict the resilience scores of individuals. (b) The variance explained is shown for seven categories. (c) Model factors weights projected to the resilience scores. Error distribution estimated across 1000 bootstrap samples are shown.

Our analysis confirmed that individuals in the UKB cohort who self-reported high resilience effectively coped with adversity, showing consistency over a 16-year follow-up. Additionally, these resilient individuals were more likely to recover from psychological symptoms over time. We also identified blood immune biomarkers that may underlie their better response to trauma. These findings suggest stabilized resilience in this group, providing a foundation for further exploration of its psychosocial mechanisms. Thus, we further investigated the relationship between participants’ self-reported resilience scores at FU2 and their individual, familial, and community-level characteristics at baseline using a nonlinear iterative partial least squares (NIPALS) regression algorithm (Fig 4a). The model explained a total of 23.98% of the variance in the resilience score in the validation dataset. The model-predicted resilience score could accurately reflected individuals’ resilience performance in the validation dataset, as evidenced by significant trauma × predicted resilience interactions attenuating trauma’s effect on affective disorders at baseline, FU1, and FU2 (*P*_*two-tailed*_ < 0.05, **Supplementary Table 34–36**), and greater PHQ-9 and GAD-7 score reductions from FU1 to FU2 in individuals with high predicted resilience (*P*_*two-tailed*_ < 0.05; **Supplementary Table 37–38**). Moreover, we found the most explained variance coming from personality (19.00%), subjective satisfaction (8.26%), and social support (5.08%) in the model, whereas economic and lifestyle explained the least variance (<2%) (Fig 4b, detailed feature weights in the model see Fig 4c, **Supplementary Table 32–33**).

### Generalization of resilience model in adolescent cohort

A recent study challenged the enduring protective effects of adolescent resilience when defined solely by the absence of psychiatric diagnoses (i.e., outcome-based resilience), potentially due to developmental dynamics^9^. To investigate if our proposed resilience model could address this challenge, we analysed longitudinal data from 692 IMAGEN cohort participants (mean age 13.94 years [SD 0.49]; 383 [55.3%] female) with complete childhood trauma histories, psychosocial assessments, and DAWBA data from baseline (age 14) and all follow-ups (ages 16, 19 and 23). The cohort included 347 individuals (50.1%) meeting trauma-group criteria (≥2 adversity types) at baseline.

**Figure 5:**
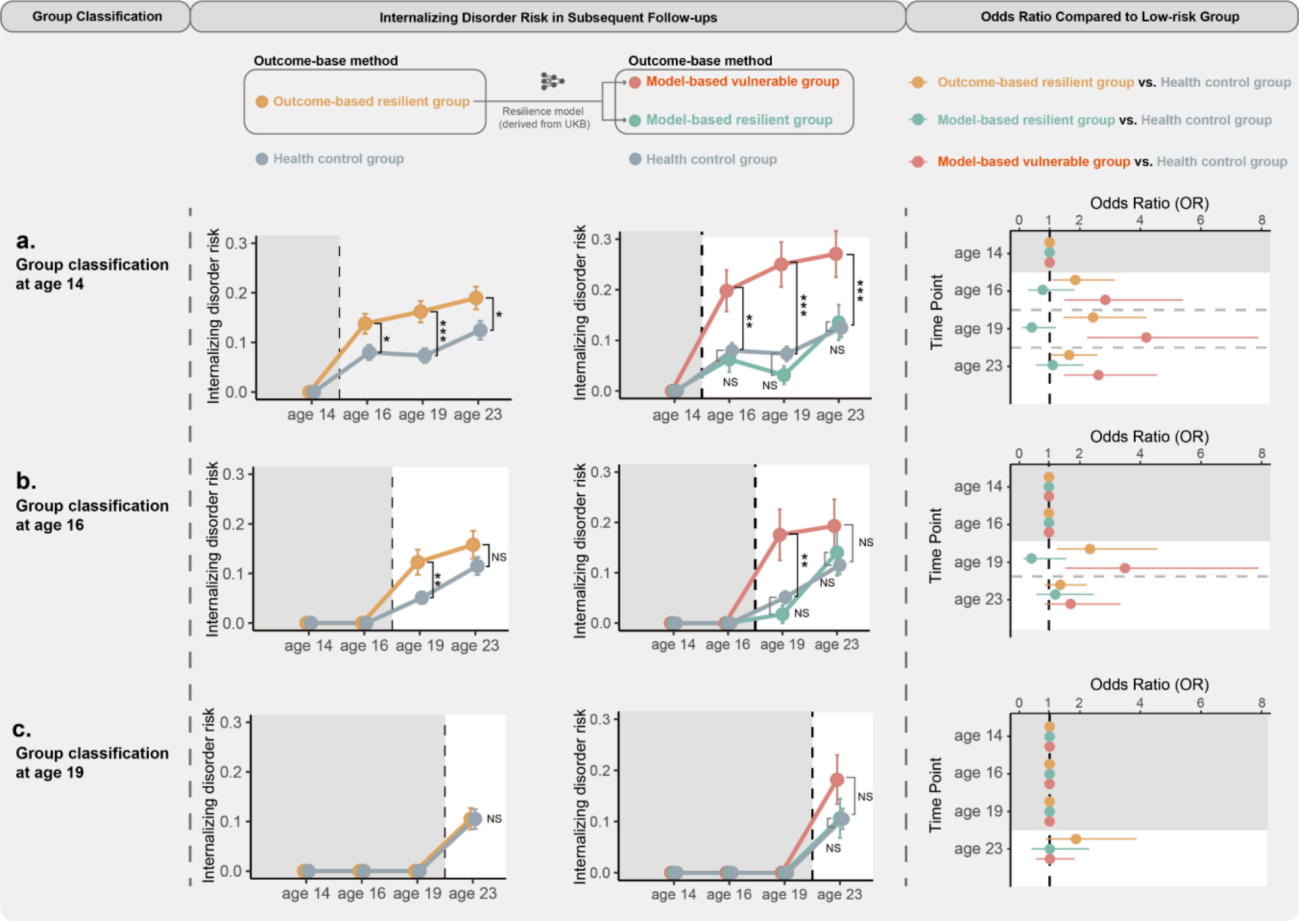
Comparing model-based and outcome-based approaches for resilient outcome prediction (a) Left: Line plot showing incidence rates of internalizing disorders at ages 16, 19, and 23 for groups categorized at age 14 using outcome-based and model-based methods, compared to the health control group (error bars: mean ± SE; *p<0.05, **p<0.01, ***p<0.001). Right: Plot of odds ratios comparing each group to the health control group (error bars: 95% CI). (b) As in (a), but for groups categorized at age 16, with incidence rates at ages 19 and 23. (c) As in (a), but for groups categorized at age 16, with incidence rates at age 23.

Conforming with previous observations, we first showed that outcome-based resilient participants (defined as having no psychiatric diagnoses at age 14 despite childhood trauma exposure, N = 290) remain at significantly higher risk of internalising disorders at subsequent assessments (FU1 [age 16], FU 2 [age 19], and FU 3 [age 23]) compared to the health controls (no trauma exposure and no diagnoses, N = 313; Figure 5a, **Supplementary Table 39–41**))^9^. A similar pattern was observed when resilient participants were alternatively defined at FU1 (age 16), highlighting the limitation of an outcome-based resilience definition.

Remarkably, of the 290 outcome-based resilient participants, our model-based resilient (top 33%, N = 96) subgroup showed no increased internalising disorder scores from BL to FU3 (age 14–23) compared to the health controls (non-inferiority test, FU1: OR = 0.76, 95% CI = 0.28 to 1.82], *P*_*two-tailed*_ = 0.56, *P*_*non-inferiority*_ = 0.004; FU2: OR = 0.41, 95% CI = 0.09 to 1.20], *P*_*two-tailed*_ = 0.15, *P*_*non-inferiority*_ = 0.0006; FU3: OR = 1.10, 95% CI = 0.54 to 2.11], *p*_*difference*_=0.78, *p*_*non-inferiority*_=0.014)). In contrast, the model-based vulnerable (bottom 33%, N = 96) subgroup showed higher internalising disorder scores compared to both the model-based resilient participants (FU1: OR = 3.70, 95% CI = 1.48 to 10.57], *P*_*two-tailed*_ = 0.008; FU2: OR = 10.33, 95% CI = 3.44 to 44.69], *P*_*two-tailed*_ = 0.0002; FU3: OR = 2.37, 95% CI = 1.15 to 5.09], *P*_*two-tailed*_ = 0.02; Fig. 5a) and health controls (FU1: OR = 2.84, 95% CI = 1.47 to 5.41], *P*_*two-tailed*_ = 0.0016; FU2: OR = 4.20, 95% CI = 2.24 to 7.91, *P*_*two-tailed*_ < 0.0001; FU3: OR = 2.6, 95% CI = 1.47 to 4.56], *P*_*two-tailed*_ = 0.0008; Fig. 5a). Consistent findings were also held when our model-based resilience was defined at other time points (Fig. 5b, c, **Supplementary Table 42**). Noteably, reidentifying model-based resilient groups using a model only with personality traits would essentially retain the above findings (**Supplementary Fig. 3**, **Supplementary Table 43–46**), thus highlighting personality’s prominent role in adolescent resilience.

## Discussion

Using UK Biobank (UKB) data (N = 164,929), we developed a psychosocial model based on valid self-reported resilience scores, highlighting personality and subjective satisfaction as core determinants. Crucially, this model successfully predicted long-term mental health outcomes in trauma-exposed adolescents (superior to the outcome-based traditional approach), demonstrating lifespan consistency in the underlying psychosocial mechanisms of resilience. These findings unveil new opportunities for targeted early interventions in at-risk populations.

Personality traits (e.g., the Big Five in the present study) consistently correlate with adult resilience, but their interaction with external factors like family and community support remains unclear^19,26–28^. Also, subjective satisfaction, reflecting personal appraisal, outweighs external factors in driving resilience^29^. In our exploratory analyses, resilient individuals reported higher satisfaction than vulnerable peers under similar circumstances (**Supplementary Table 47, Supplementary Fig. 4**), suggesting a positive thinking style amplifies perceived external conditions’ impact. in line with this, recent research indicated that a positive thinking style facilitates a more optimistic view of external circumstances, thereby enhancing an individual’s capacity to manage stress effectively^30,31^. Moreover, previous research has shown an association between individuals’ appraisal style and their personality traits, suggesting that personality traits may shape subjective perceptions, thereby influencing the impact of the external environment on resilience^32^. These complex interactions among personality, subjective perceptions, and environment highlight the need for further mechanistic studies.

Adolescence is a period marked by rapid fluctuations in resilience, which may stem from changes in the external environment^9,33–36^. For instance, trauma-exposed adolescents may temporarily maintain mental health under supportive conditions, but changing circumstances can resurface trauma’s negative effects^33^. This instability, therefore, hinders accurate identification of resilient adolescents using the otherwise powerful outcome-based methods, as short-term resilience may not predict long-term mental well-being in adolescence, risking misclassification. Our findings confirmed this: trauma-exposed individuals without psychiatric symptoms before age 14 faced higher internalising disorder risk than non-trauma-exposed peers. While extending the observation period (i.e., defining resilience until individuals reach age 19) reduces this misclassification, it nevertheless misses adolescence as a window for timely intervention. Based on psychosocial factors identified in our model, we proposed a lifespan approach to accurately identify adolescents who can exhibit long-lasting resilient performance in their later life. Our approach, integrating multi-level psychosocial factors, effectively differentiates between those likely to demonstrate stable, long-term resilience (model-based resilient group) and those at higher risk of developing mental health issues in the future, though temporarily without psychiatric symptoms following trauma (model-based vulnerable group). This approach demonstrates the consistency of resilience factors across the lifespan and enables early, targeted interventions for trauma-exposed adolescents.

Remarkably, among the stable middle-aged and elderly population, our findings revealed that self-reported BRS scores effectively reflected their resilience when facing real-life adversities in the past. We proposed that middle-aged and elderly individuals, with their wealth of life experience, are better equipped to accurately assess the “ability to bounce back from stress” as measured by the BRS. Furthermore, our analysis of longitudinal mental health data showed that the ability reflected by the BRS is not limited to trauma-related contexts. In daily life, individuals with higher BRS scores demonstrated better mental health trajectories, highlighting the essential role of this ability in maintaining psychological well-being. Additionally, our findings reveal that resilient individuals exhibit a physiological basis that resists trauma’s effects and mitigates the relationship between trauma-affected biomarkers and affective symptoms. These blood samples, collected 16 years ago during baseline visits, indicate that the resilience measured by the Brief Resilience Scale (BRS) in this cohort reflects a stable, trait-like characteristic with enduring physiological underpinnings. These findings ensured the robustness of our psychosocial model, developed from UKB, in accurately identifying psychosocial factors related to resilience.

However, we still require more comprehensive longitudinal data to further elucidate the psychosocial factors underlying resilience. Although our study has included groups representative of two highly typical age periods—adolescence and mid to late adulthood—and has successfully demonstrated the stable psychosocial factors within these cohorts, future research will need to validate the role of these factors in resilience development within earlier age groups using longitudinal data. Meanwhile, due to the observational scope of the IMAGEN project, our observations of resilient performance in adolescents were limited to the age of 23. Subsequent studies may necessitate a longer observational period that encompasses adulthood to confirm that adolescents identified as resilient by our model are able to maintain sustained mental health over a lifelong time. It should also be noted that the ‘healthy volunteer’ selection bias in the UKB is present, where volunteers have a healthier lifestyle, better education, and more favourable economic conditions compared to the general UK population. This may lead to an underestimation of the impact of environmental factors on the development of resilience in our model.

In conclusion, we provide a lifespan approach to investigate the resilience process throughout human development. Based on the stable middle-aged and elderly population, we identified robust psychosocial factors related to resilience and evaluated their contributions using multivariate machine learning methods. This resilience model helps to address the long-lasting challenge of predicting future mental health status in post-adversity individuals during adolescence, a period characterised by rapid changes in psychological, physiological and environmental conditions. This lifespan approach offers novel insights into understanding resilience and carries substantial implications for the development of timely early interventions and the prevention of potential mental health disorders triggered by adversity.

## Methods

### Study design and participants

This population-based study used the UK Biobank (UKB), a longitudinal cohort of 503,317 adults aged 40–69 years, recruited from 2006–2010 across 22 UK centres. Baseline assessments included sociodemographic, lifestyle, environmental, early-life, mental health, and biomedical data via questionnaires, interviews, and physical examinations. Two online follow-ups (2016–2017, n=157,235; 2022–2023, n=175,236) collected recent traumatic events and mental health data. The study received ethical approval (North West Multi-Centre Research Ethics Committee, 11/NW/0382, 17 June 2011), with written informed consent from all participants. Only participants who completed the Brief Resilience Scale (BRS) at follow-up 2 and provided complete covariates (age, sex, BMI, education years, ethnicity) were included in this study.

The IMAGEN study, a longitudinal adolescent cohort (n=2,224 at baseline), assessed psychological, behavioural, clinical, and environmental factors from age 14 (baseline) to 23 (follow-up 3). Only participants (n=692) who completed all four visits and provided complete data on childhood adversity, internalised disorders, and psychosocial factors were included for analyses.

### Adversity

In UKB, recent traumatic events (e.g., serious illness and violent crimes) were assessed via checklists at baseline (in the past 2 years) and follow-ups (in the past year), as detailed in the **Supplementary Table 1**. In the present study, we count the incidence of recent traumatic events experienced by each participant at each visit and designate the count as ‘traumatic events exposure’ for subsequent analytical examinations. Long-term adverse factors included 45 variables related to potential adverse effects on mental health across eco-social (e.g., loneliness), early-life (e.g., maternal smoking), and environmental (e.g., pollution) domains, as listed in the **Supplementary Table 2**.

Childhood adversity in IMAGEN was recorded across five domains: accidents, family dysfunction, maltreatment, unstable family structure, and peer victimisation (detailed definitions were provided in the **Supplementary Table 3**).

### Mental health measures

Affective symptoms in UKB were assessed at baseline using the Patient Health Questionnaire-4 (PHQ-4), a four-item scale for depression (PHQ-2) and anxiety (GAD-2) symptoms, rated on a 0–3 Likert scale. At follow-ups, PHQ-9 (9 items) and GAD-7 (7 items), rated on a 0–3 Likert scale, were each scaled to a 10-point score and summed to derive affective symptoms scores. In IMAGEN, the Development and Well-Being Assessment (DAWBA) was used to generate DSM-5 diagnoses of internalised disorders (e.g., depression, anxiety, and PTSD) at each visit, with details provided in the **Supplementary Methods**.

### Resilience Measures and Group Classification

Resilience in UKB was measured using the BRS, a six-item self-report scale assessing the ability to recover from stress (e.g., “I tend to bounce back quickly after hard times”), at follow-up 2. Full BRS items are listed in the **Supplementary Methods**. Based on their BRS scores, participants were categorised into resilient (≥66th percentile, ≥4), moderate (33rd–66th percentile, 3–4), and vulnerable (≤33rd percentile, ≤3.33) groups (Fig. 1c).

In the IMAGEN cohort, participants were classified based on trauma exposure prior to assessment: those experiencing ≥2 types of adversity (n=347, 50.1%) were assigned to the trauma group, which was subsequently stratified into resilient and vulnerable subgroups based on psychiatric outcomes of DSM-5 diagnoses (i.e., following the outcome-based definition of resilience). Individuals with ≤1 type of childhood trauma and no psychiatric diagnoses comprised the health control group.

### Psychosocial Factors

49 psychosocial phenotypes, grouped into personality, satisfaction, early-life, lifestyle, family support, social support, and demographics, were selected as factors for resilience modelling in UKB; of which the questionnaire entries, except for the personality, are listed in the **Supplementary Table 4**. Due to the absence of a validated personality scale in UKB, personality proxies were derived from touchscreen questionnaires mapping to Big Five traits, with details in the **Supplementary Table 5**. In IMAGEN, 13 psychosocial phenotypes across similar categories were used, listed in the **Supplementary Table 6**.

### Biomarkers

In UKB, blood samples were collected at baseline from all 500,000 participants and assayed for blood count (Category 100081) and blood biochemistry (Category 17518). Blood-based proteomic data, generated by the UK Biobank Pharma Proteomics Project, profiled plasma samples from 54,219 participants (2941 proteins), with 2920 proteins analysed after quality control. Details on biomarker collection, processing, and quality control are provided in the **Supplementary Methods** and the **Supplementary Table 7**.

### Statistical Analysis

In UKB, within each of the resilience groups (i.e., resilient, moderate, and vulnerable), a linear regression model was utilized to investigate the associations of individuals’ adversity exposure with their mental health status, adjusting for covariates including age at baseline, gender, ethnicity, education, and Body Mass Index (BMI). The correlation coefficients (r values) were obtained from these linear regressions as the standardised regression coefficients. Correlation comparison was then conducted using the R package ‘cocor’ to investigate if the above associations were significantly different between the resilient and vulnerable groups^37^.

We also employed the linear regression model to examine the associations of the resilience scores of BRS with the longitudinal changes in depression and general anxiety scores from FU1 to FU2 in UKB, adjusting for covariates of age at baseline, gender, ethnicity, education, and BMI. Furthermore, given the differences in FU1 characteristics between the resilient and vulnerable groups, propensity-score matching (1:1 ratio) was employed to ensure that no statistically significant differences existed between the resilient and vulnerable groups in any of the FU1 depression and general anxiety scores, as well as other covariates.

Mediation analyses (significance assessed via 1000 bootstrapping processes) were used to explore the potential roles of blood biometrics and proteins as mediators of trauma–mental health relationships, controlling for covariates (age, sex, BMI, education years, ethnicity, and research sites). To further explore potential biological insights, enrichment analyses (Gene Ontology and Reactome pathways) were conducted on plasma proteins that exhibited significant mediation effects on the relationship between trauma exposure and affective disorders. The Benjamini-Hochberg method was applied to correct for multiple testing, with a false discovery rate (FDR) threshold of <0.05 set for statistical significance.

A nonlinear iterative partial least squares (NIPALS) regression algorithm was used to model resilience-associated psychosocial factors in 17,685 UK Biobank participants with complete data. The NIPALS algorithm was selected for its interpretability as a machine learning model, as it quantifies the contribution of each factor (x) to the resilient outcome (y), facilitating a precise understanding of the psychosocial factors underlying individual resilience. In the discovery dataset (N=10,000), we trained the model with a tenfold cross-validation setting. Notably, we reduced 49 factors into three homogeneous components based on the following optimizations: (1) maximal explained variance increase and (2) RMSE reduction inflection (elbow method). The validated model was tested in an independent cohort (N=7685). In addition, we organized the 49 factors into 7 categories to assess the model’s explained variance (R^2^) in the self-reported resilience scores for each category separately.

For the IMAGEN cohort, we conducted non-inferiority testing (α=0.05 for a one-tailed test) to compare mental health outcomes between the resilient and health control groups. The margin Δ was determined via G*Power sensitivity analyses (80% power), representing the smallest clinically meaningful effect size.

## Supplementary Files

This is a list of supplementary files associated with this preprint. Click to download.

- SupplementaryApsychobiologicalresiliencefactorformentalhealthacrossthelifespanrevised1020.docx

## Data Availability

The UK Biobank is open to bona fide researchers who can apply for access through its official website at https://www.ukbiobank.ac.uk. Additional information on the registration and application process for data access is available at http://www.ukbiobank.ac.uk/registerapply/. The IMAGEN dataset can be obtained from a dedicated database located at https://imagen2.cea.fr.

## Code availability

The code that supports the findings of this study is available on GitHub at https://github.com/yangzhengyu1017/Lifespan_Resilience_model.
